# Oxidative Stress Induced Mechanisms in the Progression of Periodontal Diseases and Cancer: A Common Approach to Redox Homeostasis?

**DOI:** 10.3390/cancers2020670

**Published:** 2010-04-26

**Authors:** Mena Soory

**Affiliations:** Periodontology, King’s College London Dental Institute, Denmark Hill, London SE5 9RW, UK; E-Mail: mena.soory@kcl.ac.uk; Tel.: +0044 (0)20 3299 3057

**Keywords:** oxidative stress, cancer, periodontal disease, inflammatory profile, antioxidants

## Abstract

There is documented evidence of significant associations between cancer of the lung, kidney, pancreas, hematological and oral cancers and periodontal diseases of the supporting structures of the teeth. Enhanced lipid peroxidation, raised levels of TBARS and the oxidative stress marker malondealdehyde have been detected in breast cancer with reduced antioxidant capacity, also characteristic of periodontal diseases. Antioxidants could overcome this deficit and attenuate disease progression by down regulating glutathione detoxification/redox buffering system and inhibiting key transcription factors. Periodontal disease may be a critical marker of a susceptible immune system, or initiate cancer risk with a pro-oxidant inflammatory profile.

## 1. Introduction

### 1.1. Association between Periodontal Disease and Cancer; Case Definition of Periodontal Disease

Severe forms of periodontal diseases impose a significant inflammatory loading, which could impact on the progression of systemic conditions including carcinogenesis. Conversely, malignant progression of tumors has been shown to be associated with the development of oxidative stress, which highlights the relevance of antioxidants in anti-cancer therapeutics, also relevant to the adjunctive management of periodontal diseases. In this review, similarities in the progression and implications of inflammatory pathology in chronic periodontitis and cancer are considered, highlighting a role for antioxidants as adjunctive therapeutic agents. Examples that highlight similar mechanisms of progression and adjunctive agents that contribute to redox homeostasis in cancers and periodontal diseases are considered in this review. 

Periodontitis is a chronic inflammatory disease of the supporting structures of the teeth initiated by pathogenic bacteria, predominantly *Prophyromonas gingivalis*, *Tannerella forsythensis* and *Treponima denticola* [1], associated with numerous others in plaque biofilm attached to root surfaces, leading to deepening periodontal pockets and alveolar bone destruction [2]. Population based studies have used many definitions of periodontitis in the literature, which are likely to encompass several levels of involvement [3]. Periodontal parameters used to define moderate and severe periodontal disease enhance case definition and accentuate the importance of thresholds of periodontal parameters and the number of affected sites in determining prevalence; with implications on inflammatory loading. These distinctions are not often reported in the context of their association with systemic diseases. Improving oral health literacy is an important aspect of healthcare, considering the fact that most oral diseases are preventable at an early stage and relatively inexpensive when addressed at this stage, and also likely to avoid more complex progression associated with co-existing systemic diseases. Large segments of the population may be faced with multiple barriers that impede access to health care resulting in a higher prevalence of disease. Changes in the infrastructure aimed at optimizing skills of the dental workforce in reaching out to these populations would be an important aspect of improved healthcare [4]. Periodontitis is considered to be an infectious disease of the supporting structures of the teeth modulated by susceptibility and bears comparison to other chronic inflammatory diseases; in view of its association with diabetes mellitus, coronary heart disease, cerebrovascular and pulmonary diseases, low birth weight and overall mortality. There is some documentation of associations between the prevalence of periodontal disease and carcinogenesis in recent literature, underpinned by inflammatory mechanisms common to both entities. 

The relationship between periodontitis and cancer was investigated in an epidemiological follow-up study [5]. The study included 11,328 individuals in the age range of 25–74 year who were followed longitudinally after medical and dental examinations at baseline with four follow-up examinations within a 10 year period. Demographic variables included age at baseline, race, gender, marital status, education and financial status of individuals examined. Potential environmental risk factors for neoplasia were taken in to account. Examination of the periodontium comprised the extent of overt gingival inflammation, the presence of periodontal pockets with loss of attachment and tooth mobility. An evaluation of the association between periodontal status and lung cancer was performed using Cox proportional hazard models. They were fitted to assess whether individuals with gingivitis, periodontitis or edentulism were at a greater risk of fatal malignant neoplastic change in the bronchus and lung than those with a healthy periodontium over a 10 year period. Different forms of confounding variables were specified (e.g., edentulism, environmental factors) to evaluate how robust the periodontitis-cancer association is. Individuals with periodontitis had a significantly higher level of risk of death from cancer. The largest contributor was lung cancer, while there was some indication of associations with prostate, breast and pancreatic cancers. When the results were limited to never-smokers, no association was found between periodontitis and lung cancer. Gingivitis, which reflects inflammatory loading and edentulousness, showed raised levels of risk of death from lung cancer. 

Periodontits could be a surrogate marker for the effects of smoking and thus provides a link with lung cancer. Results may differ depending on models used to demonstrate these associations and the inclusion or exclusion of certain demographic variables. It is relevant that smoking imposes oxidative stress and triggers a series of events that could contribute to cell transformation, in addition to the fact that the degree of inflammatory burden from the periodontium would be an important factor in order to have a systemic impact as is the case with periodontitis and other systemic associations.

### 1.2. Inflammatory Loading Imposed by Chronic Periodontitis and Predisposition to Carcinogenesis

Chronic inflammation affecting the periodontium results in the release of a steady stream of cytokines, prostaglandins, bacterial toxins and enzymes from host and bacterial cells, which have a damaging effect on the tissues, resulting in the formation of a periodontal pocket associated with destruction of the supporting alveolar bone [6]; aggressive and chronic forms of periodontitis being the commonest cause of tooth loss in adults. There is growing documentation of chronic inflammatory periodontitis as a risk factor for premature death [7] from several causes. A recent investigation evaluated the cause of death in 3273 randomly selected subjects between the ages of 30 and 40 years over a 16 year period. It was concluded that young persons with periodontal disease and missing molars were at increased risk of death from neoplasms, circulatory and digestive disorders. In a large population of well-characterized US male health professionals with self-reported periodontal disease and alveolar bone loss, an increased risk of cancer was demonstrated by Michaud and co-workers [8]. This study demonstrated a significant association between periodontal disease and hematological, renal and pancreatic cancer. These findings were reported in never-smokers. An association between periodontal disease and pancreatic cancer was reported in their previous study in the same population of health professionals [9]. It was concluded that mechanisms associated with systemic inflammatory burden and increased levels of carcinogenic compounds generated in response to periodontal pathogens and sequelae of inflammatory mechanisms, could contribute to the development of cancer. A recent case-controlled study is suggestive of a possible link between chronic periodontal disease and cancer of the tongue [10]; while other investigators are not certain of a conclusive association between periodontal disease and cancer [5]. Variations in study design, inflammatory status of the periodontium, case definition and interpretation of results could account for differences. More investigations are required prior to definitive conclusions being made regarding such links.

The progression of inflammatory periodontal disease in response to periodontal pathogens and mechanisms that trigger coexisting systemic diseases and cancer could have important implications on public health; promoting early detection, diagnosis and management. This highlights the importance of making specific case definitions of periodontal diseases for use in population studies, considering the range of periodontal diseases that can lead to tooth loss and inflammatory status of the periodontium at the time of examination. There are grades of progression with more aggressive forms progressing more rapidly with a relatively poor response to treatment [6]. It follows that when associations are made with systemic diseases, it would be important to categorize and specify the periodontal disease entity that one is working with [3], in view of diversity in the inflammatory burden imposed. 

Recent findings show that moderate periodontal disease is more prevalent than severe forms of the disease, with increasing risk of developing severe disease with age. However the risk of developing systemic diseases such as diabetes mellitus, cardiovascular disease and cancer also demonstrates an age related pattern. A direct association between co-existing diseases requires cautious interpretation [11]. Variables such as consumption of tobacco, alcohol; personal factors such as nutrition, stress, socioeconomic status, immune status and body mass index make it difficult to obtain clear correlations. Some considerations regarding periodontal disease, such as the relevance of disease aggression, potential for inducing a systemic inflammatory burden and systemic complications; and whether or not all forms of periodontal diseases would predispose individuals to the risk of systemic disease [12], are important. A pertinent point in this context may well be related to volume with regard to size of the inflammatory burden, based on disease severity, number of teeth affected and inflammatory status at the time of examination. For example, very aggressive or severe periodontal disease may be limited to fewer teeth and mechanisms associated with the consequence of excessive inflammation may not lead to significant manifestations from a limited number of sites. In addition to local mechanisms triggered as a consequence of the severity of periodontal disease, the size of the inflammatory burden would also be important.

**Figure 1:**
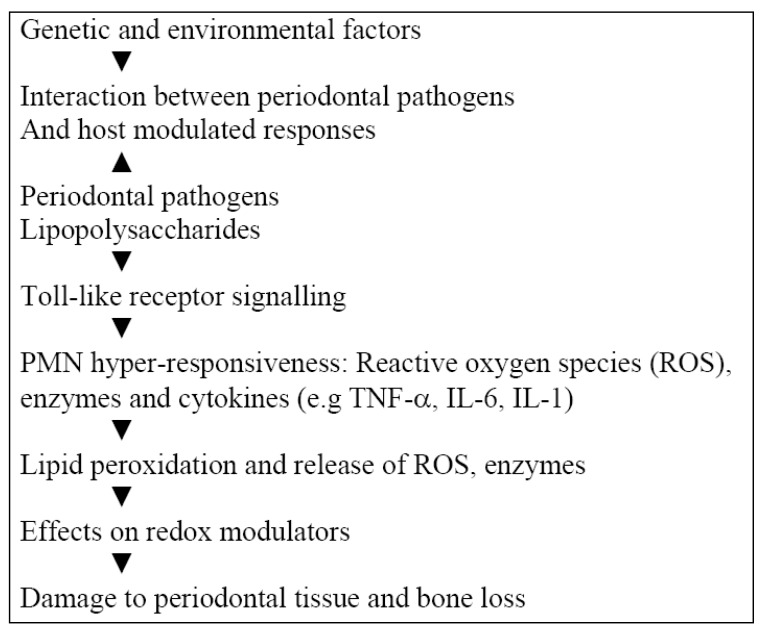
Role of host modulated inflammatory responses in destructive periodontal disease.

Some findings relating periodontal diseases to systemic conditions correlate with a more generalized distribution of very aggressive forms of periodontal diseases. However, these findings may not apply to individuals with less aggressive forms of periodontal disease or those limited to a few sites of disease activity. Thus, more research is needed to specifically qualify different types of periodontal disease, their association with systemic diseases such as cancer, and a possible causal role. Suffice it to say that a large inflammatory loading attributed to periodontal disease could correlate with cancer risk, by virtue of the mechanisms involved leading to oxidative stress induced damage to tissues (Figure 1). The management of periodontal disease also has implications on inflammatory loading thus imposed. Disease susceptibility could be influenced by predetermined factors such as genetic polymorphisms.

### 1.3. Oral Lesions and Immunosurveillance

The rare oral mucosal disorder proliferative verrucous leukoplakia (PVL) could affect the gingivae. It has a high rate of progression to squamous cell carcinoma and verrucous carcinoma. A follow up study was conducted on a group of patients with PVL affecting the mucosa of the alveolar crest and its malignant transformation. The control group, consisting of patients affected by oral carcinoma without proliferative verrucous leukoplakia, was compared with the test group with PVL for malignant transformation [13]. PVL lesions were observed most frequently on the alveolar crest with gingival involvement in 46.8% cases. Malignant progression was seen in 40.4% of patients, the alveolar crest being the most affected area. Patients with PVL were more likely to develop verrucous carcinoma (odds ratio = 6.61; 95% confidence interval) than squamous cell carcinoma (odds ratio = 0.15; 95% confidence interval) compared with controls, and with a greater incidence of cancer on masticatory mucosa, specially the gingivae and hard palate. The importance of awareness of PVL amongst periodontists is emphasized, with regard to rapid growth of verrucosity, area of ulceration, redness, induration and staining positive to toluidine blue pointers to malignant transformation with a high prevalence seen in gingivae and masticatory mucosa.

Oral diseases and cancer may have a common denominator with breakdown of immunosurveillence affecting any part of the body. There is some advantage in oncologists working with dental specialists in cases of cancer of the oral cavity [8,9]. It would be relevant to categorize periodontal disease at the time of cancer diagnosis. This poses interesting dimensions for medical oncologists being familiar with periodontal diagnosis in terms of disease aggression at the time of diagnosis and cancer outcome with varying aggression of periodontal diseases. Patient care could be improved with close liaison between oncologists and periodontists. 

Having established the co-existence or possible cause and effect relationships between periodontitis and carcinogenesis, this review addresses common ground for progression of periodontal disease and cancer based on inflammatory mechanisms that could fuel progression. Mechanisms of action of anti-inflammatories/antioxidants as potential adjuncts to treatment in order to attenuate the process are also discussed. 

## 2. Mechanisms Involved

### 2.1. Implications of Helicobacter pylori (H. pylori) in Periodontal Disease, Oral and Gastric Cancer

*H. pylori* has been implicated in gastritis, peptic ulcers and considered to be a risk factor for gastric cancer. Periodontal pockets in individuals with periodontal disease may harbor *H. pylori*. A study was done to determine whether the presence of *H. pylori* in dental plaque correlated with gastric involvement in patients with or without periodontitis [14]; 65% of patients were found to be positive for *H. pylori* in dental plaque and 50% harbored the organism in their stomach. Patients with periodontitis demonstrated a significantly higher percentage of *H. pylori* in plaque (79% *vs*. 43%) and in the stomach (60% *vs*. 33%) than in periodontally healthy patients (p < 0.05). The co-existence of *H. pylori* in both dental plaque and the stomach was detected in 78% of patients, indicating that the oral cavity could be a potential reservoir for transmission of and re-infection with *H. pylori*. 

*H. pylori* gastric infections are prevalent universally and could contribute to serious medical problems, ranging from gastritis and its sequelae to gastric carcinoma or lymphoma. Current studies indicate that *H. pylori* are present in dental plaque, although the number of organisms in individual samples is low with variation across sites within the same mouth. Gastroesophageal reflux could determine the sporadic presence of this organism in plaque. Whether the numbers of *H. pylori* present in dental plaque found in most mouths is a sufficient source of infection or re-infection for gastric conditions needs clarification [15]. It would seem logical that an improvement in standard oral hygiene procedures could complement conventional therapy of gastric ulcers, especially in cases where gastric infection has remained incalcitrant. In patients sensitized *via* gastric colonization and mucosal attachment, *H. pylori* may also be a cofactor in the recurrence of aphthous ulceration.

There is an association between *H. pylori* and development of peptic ulcers and gastric cancer [16,17,18,19,20]. *H. pylori* have also been isolated from the subgingival biofilm of subjects with chronic periodontitis and poor oral hygiene [21,22,23,24,25,26]. The prevalence of *H. pylori* in subgingival biofilm has been reported in 38% of individuals with periodontitis [24] and others have reported figures of 41% [22] and 26.6% [26]. Failure to eliminate the species from oral niches could lead to recolonization and reinfection in the gastric mucosa. More than 50% of the global population carries *H. pylori* infection. The mode of transmission could be oral-oral and gastric-oral routes [27]. The primary extra-gastric reservoir for *H. pylori* has been reported to be the oral cavity [28,29,30,31,32], although other workers have not found it to be a significant source. It raises the question whether the mouth could be a common source for re-infection of the stomach after treatment [33] and oral health status could influence its colonization and recurrence [25].

The role of the oral cavity in its transmission, particularly in the presence of periodontal disease requires clarification; although the oral cavity and dental plaque act as reservoirs for *H. pylori*. The presence of *H. pylori* in subgingival biofilm and saliva of subjects with periodontitis was evaluated by PCR [34]. Samples were obtained from 56 periodontally healthy subjects and 169 with chronic periodontitis. DNA was extracted from the samples and subjected to PCR using primers for detection of *H. pylori*; it was detected in 24% of all samples examined with a higher prevalence in subgingival biofilm samples (33.3%) compared with salivary samples (20%); (p < 0.05). *H. pylori* was detected significantly more frequently in salivary (23.5%) and subgingival (50%) samples of subjects with periodontitis than from healthy subjects (7.3% and 11.4%, respectively, p < 0.05). In subjects with chronic periodontitis *H. pylori* was detected frequently, suggestive of colonization by this species when periodontal pocketing and inflammation are present. 

The rich environment of the periodontal pocket could favor the colonization of *H. pylori*. Progressive chronic inflammation and the diversity of periodontal pathogens could provide a range of nutrients and binding sites for *H. pylori*. *Fusobacterium* species have been reported as key microorganisms in initiating co-aggregation amongst genera of initial colonizers of the plaque biofilm and also with *H. pylori* [35], amongst other pathogens in subgingival plaque biofilm. The sequence of development of plaque biofilm leads to colonization by strict anaerobes. Production of formate and fumarate are used as energy sources by species of *Campylobacter* and *Wolinella* [36] contributing to their sustenance; colonization by *Fusobacterium* may favor the establishment of *H. pylori* in the periodontal environment. An urea rich subgingival environment is likely to be selective for *H. pylori*, being a urease producing microorganism. Antagonistic mechanisms against *H. pylori* could also occur, such as the production of bacteriocin-like inhibitory proteins produced by *Streptococcus*, *Actinomyces* and *Prevotella* species [37]. It is relevant that both periodontal disease and *H. pylori* infections may be influenced by similar risk factors such as age, gender, ethnicity and socioeconomic status [38,39]. Even after adjusting for these sociodemographic variables, a significant association between the prevalence of *H. pylori* and severe periodontal disease has been reported [21]. The association between subgingival biofilm, severe periodontal disease and *H. pylori* infection is a pertinent one with regard to the possible influence of periodontal status on gastric mucosal inflammation and carcinogenesis. Links between *H. pylori*, cancer and periodontal disease have been documented; patients with the latter are more likely to test positive for *H. pylori*. 

Lesions resulting from betel quid could predispose to colonization by *H. pylori*. A case controlled study was conducted at a cancer center, to determine the presence of antibodies to *H. pylori* in betel chewers and non-betel chewers amongst those with and without oral cancer. Biopsies were cultured under microaerophilic conditions to isolate *H. pylori* [40]. Amongst the 53 oral cancer patients examined, 22.7% of betel chewers and 44% of non-betel chewers were found to be positive for IgG antibody against *H. pylori*. In the healthy group, 16.7% of betel chewers were serum positive for *H. pylori*, while none of the healthy non-betel chewers tested positive. Serological testing showed that 26.4% of oral cancer patients were positive for *H. pylori*. The presence of *H. pylori* in betel chewers with or without cancer was significant when compared with non-betel chewers. It was concluded that there was a significantly greater proportion of *H. pylori* in betel chewers in comparison with non-betel chewers, but not between those presenting with or without oral cancer; suggestive of a predisposition to colonization by *H. pylori* in the digestive tract through swallowing betel quid or during chewing, rather than a predisposition to cancer as a result of betel quid.

**Figure 2:**
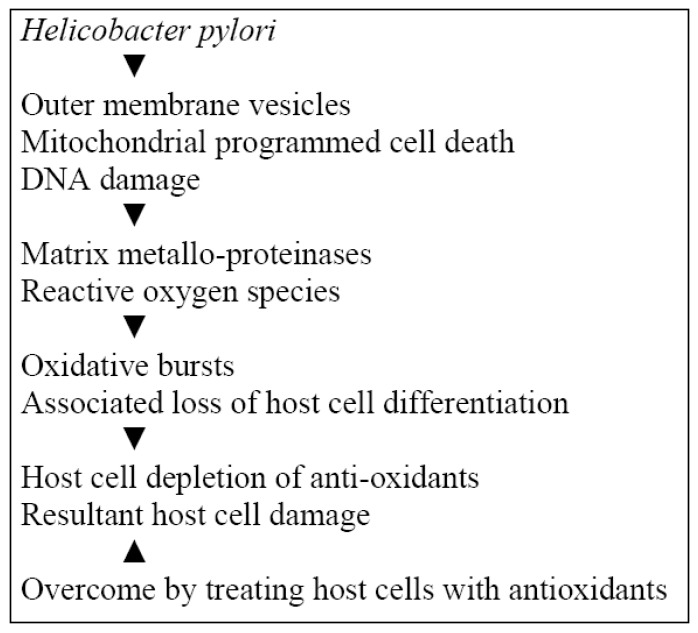
*H. pylori,* oxidative stress and carcinogenesis.

The role of *H. pylori* infection in the progressive cyto-histological differentiation associated with gastric carcinoma has been established; along pathways leading to production of reactive oxygen species (ROS) and oxidative damage to DNA (Figure 2) in combination exogenous and endogenous factors [41]. The induction of a systemic matrix metallo-proteinase (MMP) response was demonstrated in response to *H. pylori* with release of MMP-8 and other PMN degranulation products [42]. This reflects accelerated proteolysis and oxidative stress with possible extra-intestinal sequelae affecting oxidative stress driven diseases. The effects of outer membrane vesicles of a toxigenic strain of *H. pylori* were investigated in human gastric epithelial cells. There were resultant changes indicative of oxidative stress induced genomic damage associated with glutathione loss and overcome by increasing intracellular glutathione levels [43]. Mitochondrial damage induced by *H. pylori* was investigated in gastric epithelial cells. There were oxidative bursts with raised intracellular superoxide levels and mitochondrial damage consistent with a mitochondrial-ROS mediated apoptotic pathway, which was reversed by treating infected cells with the antioxidant vitamin E [44].

### 2.2. Oncogene c-Src and Cancer Invasion

The proto-oncogene c-Src is involved in cancer invasion in its inception and progression. Its impaired function could decrease osteoclastic activity and c-Src may serve as a suitable target for cancer treatment, associated bone metastases and osteoporotic diseases [45]. A dually active inhibitor of c-Src and Bcr-ABL in preventing bone resorption has been tested in early clinical trials with promising results. c-Src is a proto-oncogene involved in the genesis of and invasion by many cancers. This non-receptor tyrosine kinase also plays a crucial role in bone homeostasis, since inhibition or deletion of c-Src impairs the function of osteoclasts, the bone resorbing cells. It is thus conceivable that c-Src could be a suitable target for the pharmacological treatment of cancers, skeletal metastases and osteoporosis. The pyrrolo-pyrimidines CGP77675 and CGP76030 proved to be effective in preventing bone loss in animal models, while the effect of AZD0530, a dually active inhibitor of c-Src and Bcr-ABL, on bone resorption, has been tested in Phase I clinical trials with promising results. As far as metastatic bone disease is concerned, c-Src inhibitors could potentially have inhibitory effects both on osteoclasts and on tumor cells, and could disrupt the vicious circle established between these cell types in the bone microenvironment. In accordance with this idea, CGP76030 is able to reduce the incidence of osteolytic lesions and of visceral metastases, and to suppress morbidity and lethality in a bone metastasis mouse model without obvious adverse effects. The purine-based c-Src inhibitor AP23451 and the dual c-Src/Abl inhibitors AP22408 and AP23236 proved efficacious in reducing bone metastases in preclinical studies. In view of the key role of Src in migration, invasion and other tumor progression-associated events such as mitogenesis in many human cancers, inhibitors that target Src are regarded as promising therapeutic agents in cancer therapy [46]. These results open a new avenue for the development of innovative therapies for the treatment of bone metastatic disease. 

Extra-cellular proteases are implicated in many pathophysiological processes and their differential expression in diseases such as cancer, cardiovascular disease, pulmonary and periodontal diseases. There may be a significant increase in therapies based on extracellular protease inhibition [47]. Recent developments in targeted drug therapy for matrix metalloproteinases, serine proteases and cysteine proteases have been addressed. Proteolytic processes are required for physiological turnover. Failure in biological control of proteolysis could result in diseases such as carcinogenesis, enabling tumor invasion and metastasis associated with the passage of malignant cells through cell membranes. Protease inhibitors prevent tumor growth and dissemination of cancer cells [48]. Low molecular weight inhibitors of matrix metallo proteases, lysosomal and other proteases which inhibit protease activity show promise as anti-cancer agents and in preventing metastasis. 

### 2.3. Actions of Integrin αvβ6 in an Inflammatory Model

Over the past decade, several functions have been associated with increased expression of αvβ6 integrin such as promotion of cell migration, control of cell proliferation, activation of TGF-β, mediation of cancer cell invasion, suppression of apoptosis and modulation of protease activity. It is difficult to speculate on which of these is active when αvβ6 is upregulated *in vivo*. The findings of *in vitro* analysis have been reviewed [49]; αvβ6 is known to enhance migration and invasion. For example, it promotes keratocyte migration over fibronectin and vibronectin [50], which could be enhanced by hepatocyte growth factor and modulated *via* a pathway involving protein kinase C. Binding to fibronectin upregulated secretion of the proenzyme form of type iv collagenase, MMP-9; similarly TNF-α-dependent upregulation of αvβ6 also increased migration and MMP-9 secretion [51]. Activation of TGF-β occurs *via* αvβ6 integrin. Defects in TGF function are associated with pathological conditions including autoimmune disease, tumor cell growth, fibrotic conditions leading to scarring, as seen in pulmonary and renal fibrosis. The switch from αvβ5 to αvβ6 that seems to occur naturally when keratocytes undergo malignant transformation has been investigated [52]. It is relevant that αvβ6 is not expressed constitutively in healthy epithelia but upregulated during tissue remodeling, which occurs in wound healing and carcinogenesis [53]. Molecular mechanisms leading to the expression of αvβ6 and its eventual disappearance are unclear [54,55], although there is documentation of its *de novo* but transient expression in wound keratinocytes. It is of interest that although αvβ6 (a major activator of TGF-β1) is not usually expressed in healthy epithelia, it is co-expressed with TGF-β1 in healthy junctional epithelium linking gingivae to teeth and its expression is down-regulated in human periodontal disease. An antibody blocking αvβ6 integrin-mediated activation of TGF-β1 initiated inflammatory periodontal disease in a rat model [56]. Hence, it may be protective against periodontal disease through activation of TGF-β1. 

Wound healing and carcinogenesis have several biological processes in common and carcinogenesis may be considered to be a mis-regulated form of wound healing [57]. Expression of αvβ6 has been reported in Ca of the lung, breast, pancreas, stomach, colon, ovary, salivary gland as well as oral and skin squamous cell carcinoma [53,58,59,60,61,62,63,64]. A gradual increase in expression seemed to correlate with progression of the disease. Strong expression of αvβ6 has been reported to be a prognostic indicator in colorectal cancer [60]. Its expression per se does not necessarily drive progression to malignancy [65]. It has been reported that β6 was expressed in all *in situ* and invasive carcinoma of the floor of the mouth and often more intense at the invasive front of the tumor [66]; this is consistent with the proposal that αvβ6 promotes invasion of oral carcinoma. As a major contributor to progression of oral cancer, it should be considered as a potential therapeutic target. This is discussed in the section under adjunctive treatment modalities. 

### 2.4. Regulation of αvβ6 Expression

Mechanisms involved in the regulation of αvβ6 expression include removal of the terminal 11 amino acids resulting in mutation of the β6 cytoplasmic domain, preventing the upregulation of αvβ6 in response to cell density. This is replaced by upregulation of αvβ5, suggestive of a link in their actions by switching expression from one to the other [67]. It has been reported that high cell density selectively enhanced αvβ6 expression in cells from colonic carcinoma; this was mediated *via* protein kinase [68]. Studies show that in TNF-α deficient mice, αvβ6 is upregulated by TNF-α [69]. After treatment of skin keratinocytes for 48 h with TNF-α, a seven-fold increase in αvβ6 expression was detected [70]. Bates and coworkers have reported that TGF-β worked synergistically with TNF-α to upregulate αvβ6 in colonic cells by upregulating the critical transcription factor Ets-1 within 1 kb upstream from the β6 transcription start site [61]. As both TGF-β and TNF-α are commonly detected in cutaneous and oral wounds, this could be relevant to the induction of αvβ6. However, additional research is required before such a generalization can be made. Permitting greater specificity of targeting between normal and tumor cells would be a desirable approach to cancer therapy; and αvβ6 may represent a suitable target for this treatment strategy. 

It is relevant that integrin αvβ6, which is usually not expressed in adult epithelia, is present in healthy junctional epithelium linking the gingivae to tooth enamel and down-regulated in human periodontal disease. Integrin αvβ6 knockout mice developed severe chronic periodontal disease with epithelial downgrowth on the tooth, pocket formation and destruction of supporting bone [56]. Integrin αvβ6 is usually induced during wound healing, cancer and certain fibrotic disorders; it is a major activator of TGF-β1, a key anti-inflammatory immune regulator, and they are co-expressed in healthy junctional epithelium. When integrin αvβ6-mediated activation of TGF-β1 was blocked by an antibody in a rat model of gingival inflammation, it initiated periodontal breakdown. This integrin, which is constitutively expressed in the junctional epithelium connecting the gingivae to tooth enamel, appears to play a central role in preventing the initiation of periodontitis *via* activation of TGF-β1. 

**Figure 3:**
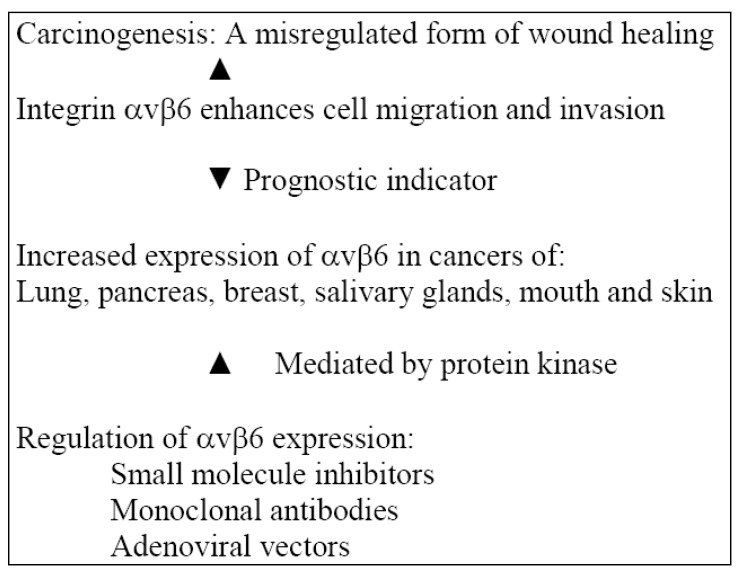
Integrin αvβ6 and its interactions in carcinogenesis.

Integrins comprise a large family of transmembrane receptors that mediate adhesion in the cell substratum. The epithelium-specific integrin, αvβ6 is a receptor for the extracellular matrix proteins fibronectin, vitronectin, tenascin and the latency associated peptide of TGF-β. The integrin αvβ6 is not expressed in healthy epithelia, but upregulated in cancer, with possible progression of carcinogenesis, and during wound healing, modulating the expression of matrix metallo-proteinases and activating TGF-β1. The role of αvβ6 integrin in cancer progression (Figure 3) has been reviewed [71]. Induction of tumor dormancy is one way of arresting tumor growth. Mechanisms that tip the balance between tumor dormancy and multistage carcinogenesis *via* immune mediated responses are poorly understood [72]. T antigen-specific CD4+ T cells are able to induce anti-angiogenic chemokines *via* TNFR1 and IFN-gamma signaling and prevent the expression of αvβ3 integrin, tumor cell proliferation, angiogenesis and multi-stage carcinogenesis, preserving T antigen-expressing pancreatic islet cells. It is relevant that in the absence of TNFR1 and IFN-gamma signaling, the same T cells are capable of promoting angiogenesis and multistage carcinogenesis. These cells are seen selectively in the tumor microenvironment around pancreatic islets where they may arrest or promote the transition of islets that are dysplastic into islet carcinomas.

## 3. Adjunctive Measures for Management and Mechanisms of Action in an Inflammatory Model

### 3.1. Treatment with αvβ6 Inhibition

The actions of integrin αvβ6 in an inflammatory model and regulation of its expression have been addressed in the above sections. Pharmaceutical companies are now developing reagents specific to αvβ6. Small molecule inhibitors for αvβ6 [73] and monoclonal antibodies against αvβ6 have also been developed [74]. There is experimental evidence for αvβ6-directed therapy [75], although data is limited. The use of viral vectors for anticancer therapy has been reviewed [76] and adenoviral vectors have been utilized for gene therapy. Adenoviruses enter cells by a combination of initial binding to a cell surface followed by integrin (αvβ3, αvβ5) mediated internalization [77]. Adenoviruses have been developed to target specific integrins [78] using molecular modifications. It may be possible to devise a similar strategy for recognition of αvβ6 and delivery of therapeutic genes for oral cancer by modification of viral vectors. 

### 3.2. Resolution of Inflammation with Lipoxins, Resolvins and Protectins

Resolution of inflammation is an important aspect of homeostasis in inflammatory diseases such as periodontal disease and cancer progression. Investigation of molecular events governing regulation of inflammation at a molecular level has resulted in the discovery of families of local-acting mediators generated from essential omega fatty acids. These groups of stereoselective chemical mediators are referred to as resolvins, protectins and lipoxins which control the duration and magnitude of inflammation; and signal resolution. Recent advances in the biosynthesis and circuit of activity of these proresolving lipid mediators are relatively novel; these concepts have been reviewed [79,80]. In view of their potent actions in human disease models, their deficiency could lead to inadequate resolution pathways and escalation of diseases; this opens new and potential avenues for resolution *via* adjunctive therapeutics along these lines. 

Lipoxins (LXs) and resolvins (Rvs) are an important consideration in the resolution of inflammation, especially in chronic disorders where the inflammatory loading from chronic inflammation could lead to carcinogenesis, for example in colonic cancer. Some of the mechanisms that trigger the formation of Rvs and LXs in the context of their role in carcinogenesis in response to inflammatory mediators have been reviewed [81]. They appear to be potent regulators of leukocytes and the production of cytokines, leading to the regulation of inflammatory sequelae and resolution relevant to carcinogenesis. Rvs are synthesized from omega-3 fatty acids eicosapentanoic acid (EPA) and docosahexaenoic acid (DHA) *via* cyclooxygenase-2/lipooxygenase (COX-2/LOX) pathways. LXs are also formed from COX-2/LOX pathways. Anti-tumorigenic effects have been demonstrated for metabolites of 15-LOX-1 and 2; and the COX-2 acetylation product 15-epi-LXA4 in response to low dose aspirin. When non-steroidal anti-inflammatory drugs like aspirin are acetylated, the mechanism for forming PGE_2_ from COX-2 which promotes tumorigenesis, switches to the synthesis of 15-epi-LXA4, which ameliorates this effect due to anti-tumorigenesis. Considering the interconnected pathways of pro-inflammatory mediators and cytokines, the anti-inflammatory properties of pro-resolving mediators need further investigation in the context of preventing carcinogenesis in response to over-exuberant inflammatory responses, which may be linked to chronic periodontitis. 

Cellular and molecular mechanisms that govern the resolution of self-limiting inflammation have been characterized [82]. Anti-inflammatory and pro-resolving properties of locally acting mediators have been demonstrated in murine resolving exudates, using lipid and protein informatics. Leucocytes in resolving exudates of murine systems are able to switch their phenotype to generate the mediators resolvins and protectins from omega-3 fatty acids There has been recent emphasis on E-series resolvins RvE1, RvE2, D series resolvins RvD1, RvD2, protectins which include neuroprotectin D1/protectin D1(NPD1/PD1) as well as epimeric forms triggered by aspirin. These agents show potent stereo-specific actions including lipoxins and play an important role in endogenous local signaling, which govern anti-inflammatory healing mechanisms. In addition to these actions, they also play an important role in enhancing microbial clearance and have protective actions in several organ systems. Considering their far reaching actions affecting host-directed antimicrobial actions, resolution of inflammation and anti-fibrotic effects, these novel chemical agents have tremendous potential in the therapeutics of diseases associated with uncontrolled inflammation and resulting oxidative stress, applicable to cancer therapy and adjunctive management of periodontal disease. 

Working out the circuits of the novel lipid-derived mediators, resolvins and protectins provides ways of interpreting the molecular basis of inflammatory diseases due to their effects on the duration and magnitude of inflammatory processes. The mode of action of these novel anti-inflammatory lipid mediators has been reviewed recently [80]. These lipid mediators were isolated from acute inflammatory murine models during spontaneous resolution. They have very potent anti-inflammatory, pro-resolving and antifibrotic actions *in vivo*, and are derived from the omega-3 fatty acids, eicosapentaenoic and docsahexaenoic acids. They could constitute a new tranche of anti-inflammatories in view of the fact that the inflammatory phenotypes seen in common diseases may well be associated with defective resolution where agonists that control inflammation could play a crucial role; while traditionally, targets for inhibition of inflammation have been the more conventional mode. Catabolism of pro-inflammatory mediators was considered to be adequate to attenuate inflammation resulting in a passive end to an inflammatory stimulus [83]. Recent documentation is suggestive of a more actively regulated process leading to homeostasis of an inflammatory process and resolution. This process involves the activity of proresolving lipid mediators, which include lipoxins, resolvins and protectins [79]. They are potent agonists, which control the duration and magnitude of inflammation. Other critical actions that restore homeostasis include uptake of apoptotic PMNs [84], clearance of mucosal surfaces [85,86], and antimicrobial defense mechanisms [87,88]. 

### 3.3. Lipoxins

The presence of lipoxins usually indicates resolution of inflammation. They are generated from arachidonic acid, an omega-6 fatty acid, and are released during the inflammatory process as a result of cellular interaction between mucosal and vascular tissue; and platelets and leucocytes [79]. Aspirin could trigger lipoxin release through the cyclooxygenase (COX) -2 pathway [89,90], by modifying the enzyme rather than acting as a conventional inhibitor. The mode of action of aspirin *via* acetylation of COX-2 affects the internal symmetry of the molecule and generates aspirin triggered lipid mediators. Amongst non-steroidal anti-inflammatory agents, aspirin is unique in being able to stimulate early formation of mediators usually seen at a later stage of healing and control of inflammation. Lipoxins play an important role in signaling macrophages [91], which phagocytose apoptotic remains of cells, being an essential component of inflammatory resolution. During an inflammatory episode pro-inflammatory cytokines such as interferon-gamma and interleukin IL-4 and IL-1β can induce the expression of lipoxins, which promote resolution and healing [92]. There are at least four generations of stable lipoxin analogs that have been shown to be effective in an inflammatory setting [93,94]. 

### 3.4. Resolvins and Protectins

Resolution phase interaction products known as resolvins and protectins are potent anti-inflammatory agents [87,85,95,96]. Resolvins, derived from the omega-3 fatty acids eicosapentaenoic acid (EPA) and docosahexaenoic acid (DHA) are known as E series and D series resolvins, respectively [97]. Aspirin triggered forms of resolvins are also produced by the COX-2 pathway; while lipoxygenase mediated mechanisms mediate their production in the absence of aspirin. Resolvins are effective anti-inflammatory and immunomodulatory agents by regulating transmigration and infiltration of PMNs at sites of inflammation [97] by decreasing cytokine expression on microglial cells [98] and by virtue of their stereospecific mechanism for enzyme inhibition which has been demonstrated. Protectins are also formed from DHA *via* a different pathway. Their anti-inflammatory and protective actions in neural tissue have resulted in being referred to as neuroprotectins [87,99]. Their actions include inhibition of PMN infiltration [95] similar to resolvins and improved wound healing in murine models [100]. These agents are very effective in low concentrations. 

An experimental murine model was used to determine the role of endogenous leukotrienes in response to an inflammatory stimulus with TNF-α [101]. As the exudates developed, the eicosanoids underwent an interesting ‘class switch’ in the lipid mediators synthesized. In place of leukotrienes which were deactivated, transcriptional regulation and synthesis of lipoxins were activated. It prevented further recruitment of leukocytes to the site concomitant with spontaneous resolution of inflammation, with the beginning signaling the end, in the context of prostaglandins [102]; resulting in a ‘non-inflammatory’ stimulation of phagocytosis of apoptotic PMNs by macrophages with monocyte recruitment [103]. 

Inflammatory bowel disease is characterized by relapsing inflammation with mucosal damage and abnormal mucosal responses [104]. Using an experimental colitis model the effects of aspirin triggered lipoxins [105] and resolvins [85] have been shown to be protective with less severe histological features of inflammatory infiltrate such as reduced PMNs and lymphocytes in response to minute amounts of resolvin in comparison with untreated mice. These therapeutic actions of lipoxins and resolvins also have potential applications in the adjunctive management of periodontitis. 

### 3.5. Efficacy of Polyphenols in Preventing Cancer

Plant-derived foods are an abundant source of polyphenols, particularly fruits, seeds and leaves. Highest concentrations of polyphenols are found in oral mucosa and the digestive tract. Monotonic intake in excessive amounts could be toxic; this could be overcome by salivary proline-rich proteins. Polyphenols and other antioxidants [106,107,108] exert preventive actions against infectious, inflammatory and apoptotic diseases *via* antioxidant activity, neutralization of human, bacterial and viral proteins and enzymes relevant to periodontal and metabolic diseases [109,110,111,112,113]. The preventive actions of polyphenols against oral disease have been reviewed from literature published over the last decade [114]. They overcome virulence of periodontal pathogens and increase the antioxidant capacity of oral fluids, suggestive of a preventive effect on periodontal disease progression. 

Well-planned human epidemiological studies, animal studies and *in vitro* investigations show good evidence of the efficacy of polyphenols in preventing oral cancer. Some of the anti-cancer effects of polyphenols are attributed to inhibition of enzymes, modulation of transcription factors implicated in carcinogenesis and mediation of receptor signaling [115,116]. Metabolites of some polyphenols could act as pro-oxidants, but anti-carcinogenic by inducing apoptosis *via* mitochondrial damage and toxicity from ROS [117], which in excess could be counter-productive and damaging. These findings are borne out in an epidemiological study which reports a direct correlation between high intake of polyphenols and colonic cancer [118]. Dietary supplementation with large amounts of single polyphenols could be damaging while a composite formulation of flavonoids is more likely to have a desirable outcome. 

### 3.6. Treatment with Green Tea Polyphenols

Several animal and *in vitro* studies have shown the efficacy of a range of polyphenols in preventing squamous cell carcinoma. Examples are mechanisms that prevent invasion and migration by inhibiting metalloproteases in response to green tea catechins [119], induction of apoptosis [120] and inhibition of cell growth [121]. 

Inhibition of colorectal carcinogenesis by green tea (GT) in azoxymethane treated mice has been investigated recently [122]. GT treatment commenced at the eighth week of age and lasted for eight weeks. Treatment with GT (0.6% W/V solution) caused a statistically significant reduction in the number of newly formed tumors, compared with water for controls (28%, p < 0.05). Green tea decreased beta-catenin levels and its downstream target cyclin D1. The expression of retinoic X receptor alpha (RXR alpha) seems to be selectively downregulated in specific mouse intestinal tumors. Although other retinoic receptors including retinoic acid receptor alpha (RAR alpha), RAR beta, RXR beta and RXR gamma were expressed in mouse adeoma, downregulation of RXR alpha appears to be an early event in colorectal carcinogenesis and independent of the expression of beta-catenin. Green tea has been shown to elevate protein and mRNA levels of RXR alpha with a significant decrease in methylation in the promoter region of the RXR alpha gene. Low concentrations of green tea appear to be effective in preventing downregulation of RXR alpha and inhibiting tumorigenesis in the mouse model. Green tea polyphenols are effective antioxidants and have beneficial actions as adjuncts in the management of periodontal diseases and as anti-cancer agents [109].

## 4. Summary and Conclusions

There are significant associations between cancer of the lung, kidney, pancreas, hematological cancers, oral cancer, especially gingival squamous cell carcinoma, metastatic cancers and progression of periodontal disease. Oral cancers could mimic the presentation of severe periodontal disease with clinical parameters of swelling and bone destruction suggestive of a common inflammatory focus in their pathogeneses. It is relevant that chronic periodontitis is associated with a small but significant overall cancer risk, which persists in non-smokers. Periodontal disease may be a useful marker of a susceptible immune system, or directly affect cancer risk as a result of inflammatory loading. A pro-oxidant status seen in severe uncontrolled periodontal diseases could contribute to a significant systemic impact with damage to organ systems distant from the focus of inflammation. The size of the inflammatory burden based on disease aggression and distribution of periodontitis have implications on the impact factor. The development of oxidative stress with malignant progression of a tumor has been reported, with evidence for the efficacy of antioxidants as anticancer agents. Common mechanisms in the progression of periodontitis and cancer, associated with a hyper-inflammatory status and a pro-oxidant profile have potential for therapeutic targeting directed at redox homeostasis. Formulation of effectively targeted anti-oxidant therapeutic adjuncts could ameliorate disease progression and minimize side effects of conventional therapeutic agents. 
